# Niche partitioning of sympatric penguins by leapfrog foraging appears to be resilient to climate change

**DOI:** 10.1111/1365-2656.12919

**Published:** 2018-12-03

**Authors:** Harriet L. Clewlow, Akinori Takahashi, Shinichi Watanabe, Stephen C. Votier, Rod Downie, Norman Ratcliffe

**Affiliations:** ^1^ British Antarctic Survey Cambridge UK; ^2^ Centre for Ecology and Conservation University of Exeter Penryn UK; ^3^ National Institute of Polar Research Tachikawa Japan; ^4^ Fukuyama University Fukuyama Japan; ^5^ WWF‐UK The Living Planet Centre Woking UK

**Keywords:** allochrony, climate change, competition, foraging ecology, leapfrog foraging, niche partitioning, penguin, tracking

## Abstract

1. Interspecific competition can drive niche partitioning along multidimensional axes, including allochrony. Competitor matching will arise where the phenology of sympatric species with similar ecological requirements responds to climate change at different rates such that allochrony is reduced.
2. Our study quantifies the degree of niche segregation in foraging areas and depths that arises from allochrony in sympatric Adélie and chinstrap penguins and explores its resilience to climate change.
3. Three‐dimensional tracking data were sampled during all stages of the breeding season and were used to parameterise a behaviour‐based model that quantified spatial overlap of foraging areas under different scenarios of allochrony.
4. The foraging ranges of the two species were similar within breeding stages, but differences in their foraging ranges between stages, combined with the observed allochrony of 28 days, resulted in them leapfrogging each other through the breeding season such that they were exploiting different foraging locations on the same calendar dates. Allochrony reduced spatial overlap in the peripheral utilisation distribution of the two species by 54.0% over the entire breeding season, compared to a scenario where the two species bred synchronously.
5. Analysis of long‐term phenology data revealed that both species advanced their laying dates in relation to October air temperatures at the same rate, preserving allochrony and niche partitioning. However, if allochrony is reduced by just a single day, the spatial overlap of the core utilisation distribution increased by an average of 2.1% over the entire breeding season.
6. Niche partitioning between the two species by allochrony appears to be resilient to climate change and so competitor matching cannot be implicated in the observed population declines of the two penguin species across the Western Antarctic Peninsula.

## Graphical abstract

Reductions in allochrony due to climate change may increase competition among sibling species. The authors investigated this in sympatric Adélie and chinstrap penguins and found that allochrony induced leapfrog foraging, substantially reducing spatial overlap compared to synchronous breeding. However, phenology of both species shifted in parallel, preserving allochrony and niche partitioning.

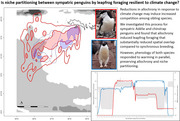

## 1 INTRODUCTION

Competition within and between species exerts strong influences over population dynamics, community structure and species distributions (Hardin, 1960; MacArthur, 1968). The potential for competition is particularly intense in communities where closely related species breed sympatrically at high densities and share limited food resources (MacArthur, 1968). Interspecific competition may be reduced by differentiating niche space along multidimensional axes such as diet (Croxall, Prince, & Reid, 1997), foraging distribution (MacArthur, 1958; Wilson, 2010) and allochrony (i.e., differences in the timing of activity among species). Allochrony in breeding phenology has been documented for a wide range of taxa (Taylor & Friesen, 2017) and can partition niches by offsetting the timing of peak resource use by competing species (Trivelpiece, Trivelpiece, & Volkman, 1987).

Animals’ breeding phenology is often timed to coincide with optimal environmental conditions, but the timing of these events is being influenced by climate change (Blois, Zarnetske, Fitzpatrick, & Finnegan, 2013). The sensitivity of breeding phenology to warming may vary between species, and the resultant uncoupling in the timing of predator demands and prey availability (“predator–prey mismatching”) have become central to our thinking about climate change impacts upon ecosystems (Parmesan & Yohe, 2003; Visser & Both, 2005). The alteration of competitive interactions by climate change has received less attention, although a growing body evidence demonstrates that the presence of competitors may have substantial effects on the magnitude and form of a species’ response to climate change. Examples include barnacles (Poloczanska, Hawkins, Southward, & Burrows, 2008), insects (Bulgarella, Trewick, Minards, Jacobson, & Morgan‐Richards, 2014), fish (Helland, Finstad, Forseth, Hesthagen, & Ugedal, 2011; Milazzo, Mirto, Domenici, & Gristina, 2013) and birds (Sætre, Post, & Král, 1999; Stenseth et al., 2015). Ecologically similar species may alter their breeding phenology in response to warming at different rates (Chadwick, Slater, & Ormerod, 2006; Lynch, Fagan, Naveen, Trivelpiece, & Trivelpiece, 2012) and, where breeding cycles become more synchronised, increases in competitive interactions may arise (Ahola, Laaksonen, Eeva, & Lehikoinen, 2007), which we hereafter term as “competitor matching.”

Seabirds are frequently used as models for the study of interspecific competition (Polito et al., 2015; Pulliam, 2000; Rosciano, Polito, & Rey, 2016), since their coloniality and central‐place foraging strategy often create high levels of competition within their shared foraging ranges (Ballance, Ainley, Ballard, & Barton, 2009; Elliott et al., 2009). Allochrony is known to reduce interspecific competition by offsetting the peak period of food demand (Barrett, Asheim, & Bakken, 1997) but also has the potential to affect spatio‐temporal overlap in foraging areas. Most families of seabird show seasonal variation in foraging ranges (incubation trips are generally longer than chick rearing ones, e.g., Barlow & Croxall, 2002; Ito, Takahashi, Kokubun, Kitaysky, & Watanuki, 2010; Kitaysky, Wingfield, & Piatt, 1999) which, when combined with allochrony, will give rise to leapfrog foraging. Leapfrog foraging has been described in high‐shore nesting oystercatchers that overfly low‐shore nesters to reach estuarine feeding habitat (Ens, Kersten, Brenninkmeijer, & Hulscher, 1992), but in the case of colonial seabirds, it would arise from the whole population of a late‐nesting species performing long incubation trips beyond the foraging range of an earlier nesting species that is performing shorter chick rearing trips. This is analogous to leapfrog migration where populations living at high latitudes overfly a mid‐latitude, resident population of conspecifics to reach their lower latitude wintering areas (Newton, 2008), albeit on smaller spatio‐temporal scales. Such behaviour has the potential to produce substantial reductions in the spatial overlap of two species’ foraging ranges compared to a situation where both species breed synchronously (Granroth‐Wilding & Phillips, 2018).

Adélie (*Pygoscelis adeliae*) and chinstrap (*P. antarcticus*) penguins (hereafter Adélies and chinstraps) are congeners that breed sympatrically across the Scotia Arc and Western Antarctic Peninsula (WAP). Here, the diets of both species are dominated by Antarctic krill *Euphausia superba,* constituting more than 95% of both species’ diet (unpublished data; British Antarctic Survey annual monitoring), and they have similar foraging behaviour (Ratcliffe & Trathan, 2012), which has prompted several studies of how niche partitioning might facilitate their coexistence (Lynnes, Reid, Croxall, & Trathan, 2002; Trivelpiece et al., 1987; Wilson, 2010). They exhibit pronounced seasonal allochrony, with Adélies initiating breeding in mid‐October and chinstraps following three to 4 weeks later (Black, 2015; Trivelpiece et al., 1987; see Lynnes et al., 2002 for diagram of phenology). This reduces competition among the two species by staggering peaks of prey demand of the two species in time (Trivelpiece et al., 1987), but its effect on partitioning foraging areas via leapfrog foraging is undocumented. Previous attempts to describe the spatial segregation between these species’ foraging distributions (Lynnes et al., 2002; Wilson, 2010) were confined to the chick rearing period and will have overestimated the degree of overlap as they assumed that the observed behaviours occurred simultaneously, when in reality they occurred 3–4 weeks apart.

The WAP is one of the most rapidly warming areas on the planet (Clarke et al., 2007; Vaughan et al., 2003), resulting in changes to chinstrap and Adélie breeding phenology (Black, 2015; Lynch, Fagan, et al., 2012) and declines in breeding numbers (Dunn et al., 2016; Forcada & Trathan, 2009; Lynch, Naveen, Trathan, & Fagan, 2012). These studies ascribed the population declines to a reduction in their preferred prey, Antarctic krill, in response to a range of factors including climate change, sea ice loss, overfishing and recovery of marine mammal populations. However, increased competition among the two penguin species for this diminishing prey resource may have further contributed to population declines, and competitor matching has been proposed as a possible mechanism for this (Lynch, Fagan, et al., 2012). An improved understanding of niche partitioning, the role allochrony plays in this and the sensitivities of these processes to climate change are therefore fundamental to understanding the drivers of population change in *Pygoscelis* penguins.

In this study, we present a behaviour‐based model of penguin foraging distributions to explore how allochrony contributes to spatial segregation in the two species. The advantage of this approach is that it takes a mechanistic approach to examining responses to changing environments, including those that have not yet been encountered by the study species (Norris, 2004). This enabled us to explore how competitive overlap might alter if the two species became more synchronous as a theoretical exercise. We then used a 20‐year time series of breeding phenology data in order to anchor the behaviour‐based model's predictions in a real‐world context and determine how niche partitioning by leapfrog foraging might be affected by climate change. We tested the following hypotheses: (a) Foraging behaviour differs between breeding stages; (b) staggering of this behaviour by allochrony will give rise to leapfrog foraging which will partition spatial niches; (c) this niche partitioning will be reduced as the degree of allochrony is shortened; (d) in areas of spatial overlap, niches will diverge along other axes such as dive depth; and (e) the two species’ phenology will advance in parallel in relation to temperature, maintaining allochrony and hence niche partitioning.

## 2 MATERIALS AND METHODS

### 2.1 Study site and tag deployments

This study was conducted at the Gourlay Peninsula on Signy Island, South Orkney Islands (60°42′S, 45°36′W), where c. 250 000 pairs of Adélies and c. 300 000 pairs of chinstraps breed sympatrically (Dunn et al., 2016). Penguins were captured in a net, after being observed leaving the nest at the end of an incubation/brooding shift or after feeding their chick. This avoided exposing eggs or chicks to predation by brown skuas (*Stercorarius antarcticus*) and ensured that all birds were breeding at the time of tag deployment. Birds were tagged between December and February of the 2007/08, 2011/12, 2013/14 and 2015/16 breeding seasons, meaning tracks were obtained from all stages of the breeding cycle (incubation, guard and crèche). Birds were fitted with both GPS loggers and time‐depth recorder (TDR) tags for between two and fourteen days in order to log their three‐dimensional foraging trips. The number of Adélie foraging trips tracked was five during incubation, 44 during guard and 18 during crèche, while those for chinstraps was 21, 89 and 7, respectively. Details of sample sizes according to species, stage and year are provided in Supporting information Appendix S1, along with justification for the relatively small samples for Adélies during incubation and chinstraps during crèche.

Specifically, devices were combined GPS‐TDR loggers (Little Leonardo GPL‐380DT, Tokyo, Japan) during 2007/08 and Fastloc2 GPS loggers (Sirtrack, Havelock, New Zealand) paired with CEFAS G5 TDRs (CEFAS Technology Ltd, Lowestoft, UK) whose clocks were synchronised in other years. Two‐part epoxy resin and waterproof tape (Tesa, Hamburg, Germany) were used to attach the GPS tags to the central back feathers and the TDR to the feathers on the rump. G5 TDRs weigh 2.7 g and have a diameter of 8 mm and length of 31 mm; Fastloc2 GPS weigh 39.9 g and measure 65 mm long, 28 mm wide and 15 mm deep; and Little Leonardo tags weigh 92 g and measure 58 mm long, 28 mm wide and 20 mm deep. The average weight of penguins fitted with devices was 3.84 kg (*SD *= 0.44) so device loads represented 2.4% (Little Leonardo) and 1.1% (F2 + G5) of their body mass. Tags of this size and placement have negligible effects on the foraging behaviour of *Pygoscelis* penguins (Ratcliffe, Adlard, Stowasser, & McGill, 2018).

Time‐depth recorders were initialised to record temperature and pressure every second in all years, while GPS tags recorded positions every second during the 2007/08 season and every 3 min in other seasons. Interruption of GPS fix acquisition by immersion resulted in actual time intervals between positions being greater than those programmed into the devices.

### 2.2 GPS and dive data processing

Dive statistics were extracted using the R package diveMove (Luque, 2016). The “filter” method of zero offset correction within diveMove (Luque & Fried, 2011) was used to define the sea surface, and a depth threshold of 5 m was used to exclude any nonforaging dive events (Kokubun, Takahashi, Mori, Watanabe, & Shin, 2010). Maximum depth and dive start time data were then extracted for each diving event. Foraging trips were demarcated by visualisation of tracks in ArcGIS 10.4.1 (ESRI, Redlands, CA, USA) to determine the approximate times the birds left and returned to the colonies. These times were further refined to the nearest minute using the temperature data from the TDR tags: A fast sharp decline in temperature indicated submersion and the reverse pattern indicated haulout.

The spatial distribution of foraging activity was examined using the locations of dives rather than using locations of raw GPS fixes, which would include positions where birds were commuting or resting at sea. We used the R package CRAWL (Johnson, 2015) to interpolate dive locations along the track based on the time at which the dive was initiated. CRAWL uses a correlated random walk model to produce predictions of the location of an animal along the simulated track at user‐defined time points. This avoids the unrealistic assumption of linear travel between GPS points and also generates error around the dive locations based on variability in the paths followed on successive simulations. We drew 100 simulated locations for each of the dives and combined these for all individuals within species and stage groupings.

Owing to small sample sizes of tracks within years, we pooled data for all years for further analysis. Annual variability in distributions and explanation of the implications of this for our findings are presented in Supporting information Appendix S1. We used adehabitatHR (Calenge, 2015) to generate kernel densities of dive locations along with their 50% and 95% isopleths. A smoothing (h) parameter of 0.06 was used in the kernel analysis, as this value was found to achieve an optima between constraining the 95% isopleth to the area that birds actually visited while smoothing their distributions within it. A utilisation distribution overlap index (UDOI) was used to quantify the overlap between species because it provides the best single measure of the degree to which two species share space by presuming that the species use space independently (Fieberg & Kochanny, 2005). Therefore, the resulting UDOI value would be 0 if there is no overlap, 1 if there is 100% overlap and the utilisation distributions are uniform, equal distribution across the area, and >1 if overlap is high and the utilisation distributions are nonuniformly distributed (Fieberg & Kochanny, 2005).

### 2.3 Statistical analysis of tracking data

Variation in foraging behaviour among species and breeding stages was investigated using the processed GPS dive locations and TDR dive depth data. The maximum distance from the colony reached during each trip was calculated using the R package move (Kranstauber & Smolla, 2016). Linear mixed effects models, fitted using the R package nlme (Pinheiro, 2016), were used to investigate differences in the average maximum distances from colony and average maximum dive depths between breeding stages and species. Models were fitted with an identity link and normal errors, and model selection was conducted using backward‐stepwise deletion and likelihood ratio (LR) tests. The global model consisted of maximum distance or maximum dive depth as the response variable, the interaction of breeding stage (incubation, guard or crèche) and species (Adélie or chinstrap) as the fixed factors and individual (with trip nested within it in the case of dive depths) as random intercept effects. Overlap of the two species maximum dive depths was quantified based on the overlap in the kernel densities of their frequency distributions (Mouillot et al., 2005).

### 2.4 Behaviour‐based model of foraging areas

Assessing the effects of allochrony on spatial overlap of the two species necessitates quantifying overlap in distributions at a daily resolution. It was not possible to design the field sampling of foraging trips in a manner that allowed this due to logistical constraints and availability of equipment. Instead, we created a virtual colony in which a predefined number of successfully breeding pairs of each species proceeded through their breeding season, making foraging trips with the frequency and characteristics for the given stage of the breeding season.

The foraging trips we collected were accurate representations of the paths those birds followed during the period of tracking, but these birds on other occasions, or other birds in the colony, would have made trips of similar characteristics (in terms of start and end points, duration, speed and tortuosity) but these would have followed different paths. Rather than sampling tracks from those observed (which would underestimate variation in paths), we generated random tracks around the observed ones using the CRAWL model. For each track, we allowed observation error (*SD *= 3.5 km during long incubation trips, 2.5 km during short chick rearing trips) around each GPS fix (except the start and end points which were fixed at the colony location). We then fitted the CRAWL model and generated 50 correlated random walk tracks for each observed trip and saved the locations of dives along each of these to an array.

For each breeding pair, we selected a date for the completion of the clutch from a distribution defined by the mean and standard deviation taken from the Results section. Birds would then complete a fixed number of long incubation trips (two for Adélies, three for chinstraps) and would then perform short incubation trips until hatching (Williams, 1995), each resampled from the appropriate array. After hatching, birds would make repeated brood‐guard trips (resampled from the brooding array) until the chicks crèched (after which trips would be resampled from the crèche array). Once the chicks reached fledging age, the simulations would begin for the next pair. This was repeated for 500 Adélie and 750 chinstrap penguin pairs, which preserved the ratio of abundance of these two species on the Gourlay Peninsula. The modelled number of pairs had no influence over estimates and was selected to optimise computing time, while ensuring the repeatability of estimates on consecutive runs. An animated visualisation of the model's process of track simulation through the breeding season is shown for Adélie penguins in Supporting information Animation S1.

We calculated the daily kernel density of dive locations for each species and their UDOI as described previously. The daily overlap values were plotted against date, and the area under curve (AUC) was calculated as an index of the amount of spatial overlap between the two species through the entire season.

The simulation model was used to investigate the degree of overlap between the two species’ kernels at the observed level of allochrony and in the absence of allochrony (by having chinstraps breed synchronously with Adélies). We also investigated changes in overlap resulting from reducing the level of allochrony in daily increments from the observed difference of 28 days to complete synchrony.

Overlap in dive depths of the resampled dive depths was investigated using kernel density analysis as for the observed data, but dives were grouped according to their degree of overlap horizontally. The horizontal groupings were overlap in 50% isopleths (core), in 95% isopleths (peripheral) and areas outside the 95% isopleth overlap (no overlap). These areas were exclusive of one another (e.g., the peripheral overlap area did not include the core overlap area contained within it).

### 2.5 Analysis of breeding phenology data

Long‐term patterns in the phenology of both species were investigated by modelling their mean annual laying dates on Signy in relation to October air temperatures. Mean October temperature was selected as the explanatory variable as it is strongly correlated with the laying dates of Adélies and chinstraps elsewhere owing to a link between air temperature, snowmelt and the exposure of nesting substrates (Lynch, Fagan, et al., 2012). Temperature data were sourced from the nearest long‐running weather station (1903 to present) at Laurie Island, South Orkney Islands (60°44′S 44°44′W) (British Antarctic Survey, 2018), which is 46 km to the east of Signy and at sea level. Trends in October air temperature with time were investigated using linear regression.

Annual mean hatching date was calculated using nest observation data collected during the breeding seasons of 1996–2015 (excluding 2010, when no data were collected). During each year, observers recorded the contents of 100 marked nests of each species every 2 days through to crèche. A binomial model was fitted using the proportion of nests containing one or more chicks as the response variable and the date in days after 1 October as the explanatory variable. This model was fitted for each species and year separately. The dose.p function in the MASS package in R (Ripley et al., 2017) was used to derive the day when 50% of nests contained one or more chicks to produce the mean hatching date for each species‐year combination. Mean laying dates were back‐calculated from the mean hatching dates by subtracting the average incubation periods for each species (35 days for Adélies and 36.4 days for chinstraps, which are relatively constant between years) (Lynch, Fagan, et al., 2012; Williams, 1995).

Changes in mean laying dates (expressed as number of days after 1st October) were modelled using analysis of covariance (ANCOVA), with laying date as the response variable, species as a factor and mean October temperature as a covariate. The annual residuals from the ANCOVA model were calculated for each species, and a Pearson correlation was used to test whether their residuals from the trends with October temperature were related. An ANCOVA was also used to model time trends in laying dates of the two species over the 20‐year study period, using year as a linear covariate.

## 3 RESULTS

### 3.1 Trip and dive metrics

**Figure 1:**
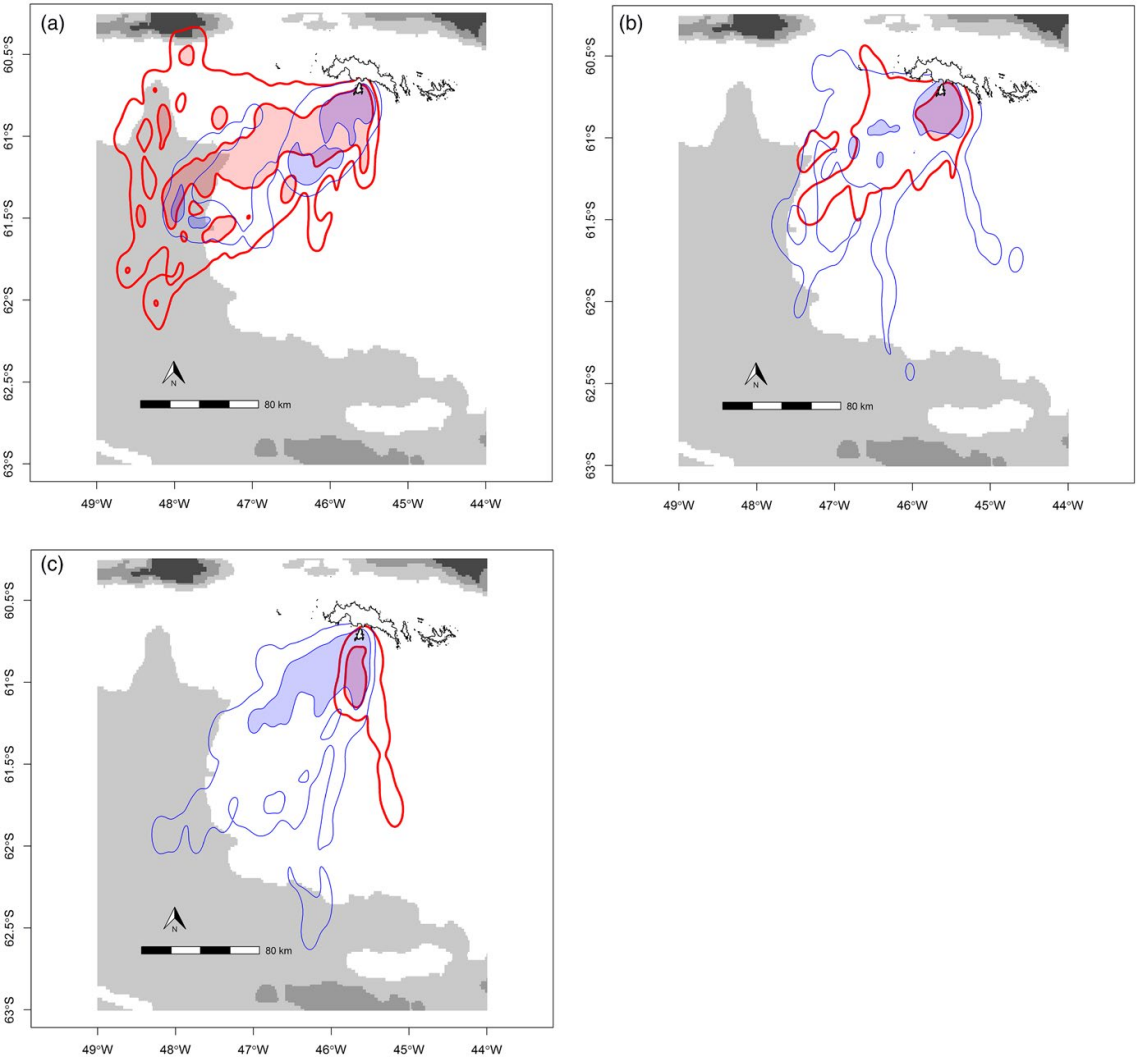
Utilisation distribution kernels of peripheral (95%) (thin line) and core (50%) foraging areas (shaded area with thick line) using raw GPS data of foraging trips during incubation (a) guard (b) and crèche (c) stage for Adélies (blue) and chinstraps (red) overlaid on bathymetry (metres) shown in greyscale shading. The maps were produced by the authors using R version 3.3.0

Incubation stage trips ranged furthest from the colony and were directed to and beyond the shelf break in a SSW direction (Figure 1a), while those during guard and crèche were shorter and occurred over the shelf within a quadrant delimited by southerly and westerly bearings from the colony (Figure 1b,c). Both species’ foraging patterns were broadly similar within breeding stages, particularly during the guard stage: Overlap of the 95% and 50% isopleths of the two species (according to naïve UDOI statistics that do not account for allochrony) was 0.493 and 0.082 during incubation, 1.968 and 0.265 during guard, and 0.227 and 0.075 during crèche, (respectively).

The maximum distances that birds travelled from their colony during a foraging trip varied according to the interaction between breeding stage and species (linear mixed effects model; likelihood ratio test LR_2 _= 13.4, *p* < 0.005). Adélie trips ranged to 75.9 km ± 19.7 during incubation and then shortened to 24.6 km ± 4.8 during brood before increasing again to 95.6 km ± 11.4 during crèche. Those of chinstraps were longer than Adélies during incubation (135 km ± 9.2) and guard (40.9 km ± 7.8) but shorter during crèche (35.9 km ± 20.21). The random between‐individual effect explained 43% of the variability in the intercept.

Dive depths were not significantly affected by the interaction of species and breeding stage (linear mixed effects model; LR_2_ = 0.53, *p* > 0.7) nor an additive effect of breeding stage (LR_2_ = 5.38, *p* > 0.05), but that of species alone was highly significant (LR_1_ = 11.37, *p* < 0.0001). Chinstraps dived deeper on average (39.4 m ± 2.6) than Adélies (25.35 m ± 3.19). The between‐individual random effect explained 33.7% of the variability in the intercept and foraging trip within individuals just 7.9%. The overlap in the frequency distributions of the two species’ dive depths across all stages was 0.77.

### 3.2 Simulated effects of allochrony on spatial overlap

**Figure 2:**
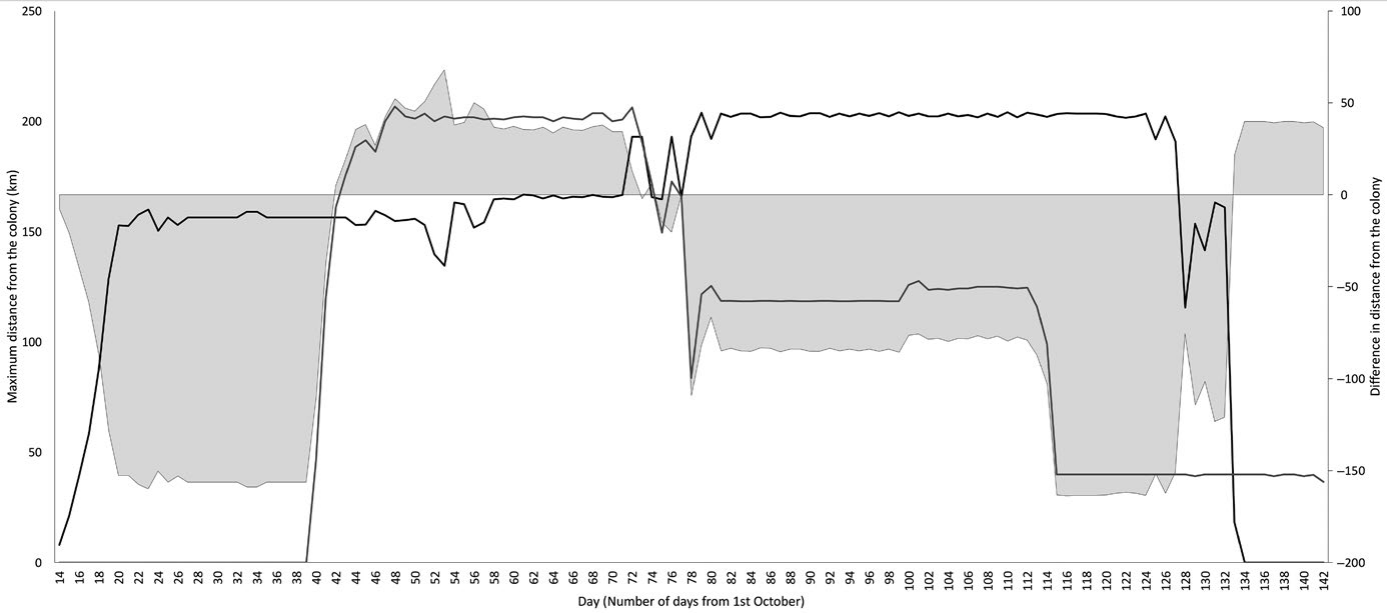
Leapfrog foraging behaviour throughout the breeding season based on Adélie (black line) and chinstrap (grey line) foraging distances. Shaded areas show when one species has leapfrogged the other by foraging further away from the colony. Areas below the dotted line show when Adélies have leapfrogged chinstraps and areas above show when chinstraps have leapfrogged Adélies (difference = daily maximum chinstrap distance—daily maximum Adélie distance)

The behaviour‐based model revealed that allochrony, in concert with the variation in trip characteristics among breeding stages, caused the two species to leapfrog each other over the course of the breeding season. Chinstraps leapfrogged Adélies by performing long incubation trips while the latter were performing short incubation and brood‐guard trips. As chinstraps began shorter brood‐guard trips, Adélies leapfrogged back over them to perform long crèche trips. Chinstraps continued short trips through the remainder of their breeding season as Adélies completed chick rearing and departed south to moult (Figure 2, Supporting information Animation S2).

**Figure 3:**
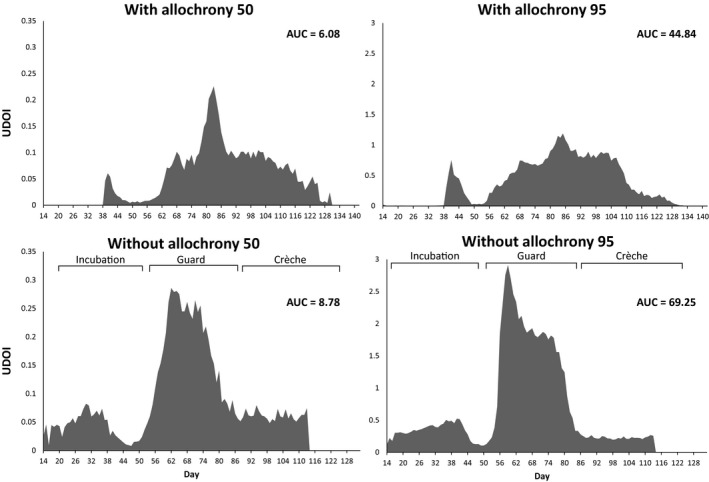
Daily (number of days from 1st October) utilisation distribution overlap index (UDOI) values, and area under the curve (AUC) values, for with allochrony (top panel) and without allochrony (bottom panel) in core (left column) and peripheral (right column) foraging areas

Theoretical scenarios showed that, in the absence of allochrony, the overlap in the AUC of all the daily UDOI values was 44.4% higher in core foraging areas and 54.0% higher in peripheral foraging areas over the entire breeding season (Figure 3). Interestingly, the level of overlap observed at the midpoint of the breeding season if birds bred synchronously was approximately double that for the observed level of allochrony: This corresponds to the guard period when parents are constrained to perform short trips that provide frequent meals for their rapidly growing chicks. We also found that if allochrony decreased by a single day, competitive overlap increased by an average of 2.1% in core foraging areas and 1.8% in peripheral foraging areas over the entire breeding season.

**Figure 4:**
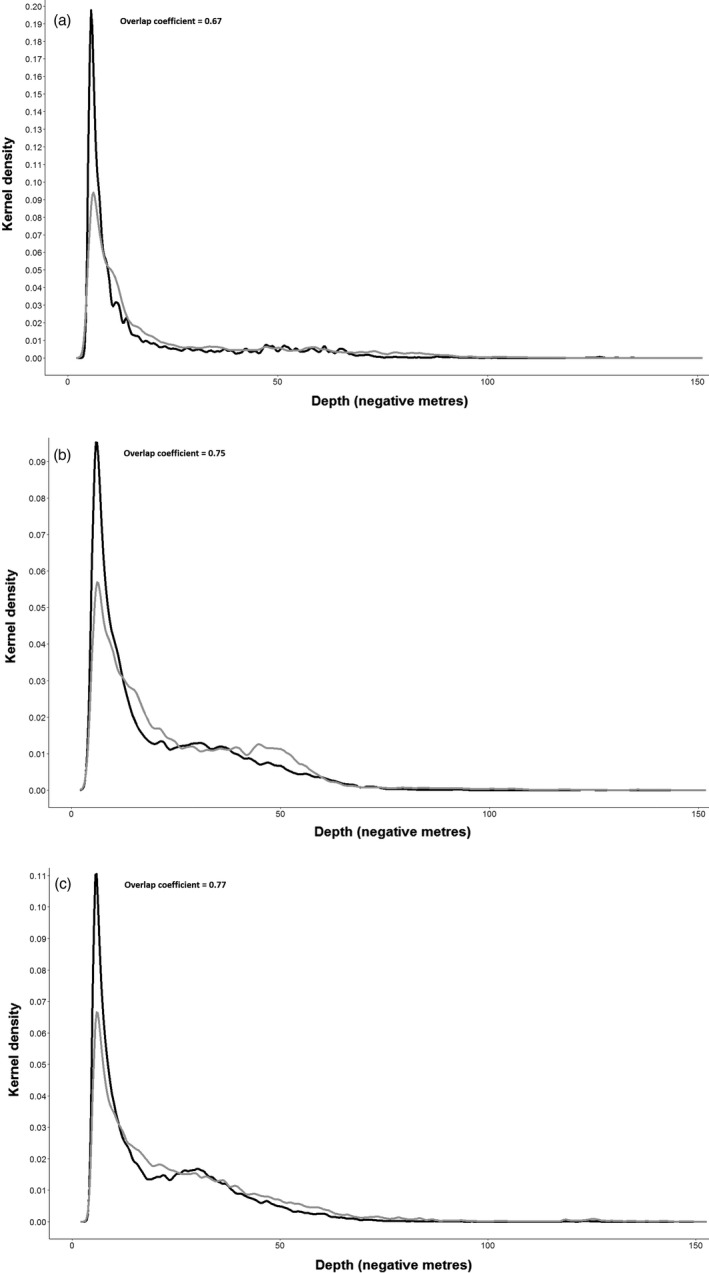
Kernel density estimation curves of vertical overlap in dive depths for core (a), peripheral (b) and no horizontal overlap areas (c) between Adélies (black) and chinstraps (grey)

The kernel overlaps in dive depth frequency distributions differed according to the degree of horizontal overlap. Overlap values were 0.75 and 0.77 in areas of peripheral and no horizontal overlap, but were lower at 0.67 in core foraging areas due to Adélies performing a greater proportion of their dives at shallower depths (Figure 4).

### 3.3 Timing of breeding phenology in relation to October air temperature

**Figure 5:**
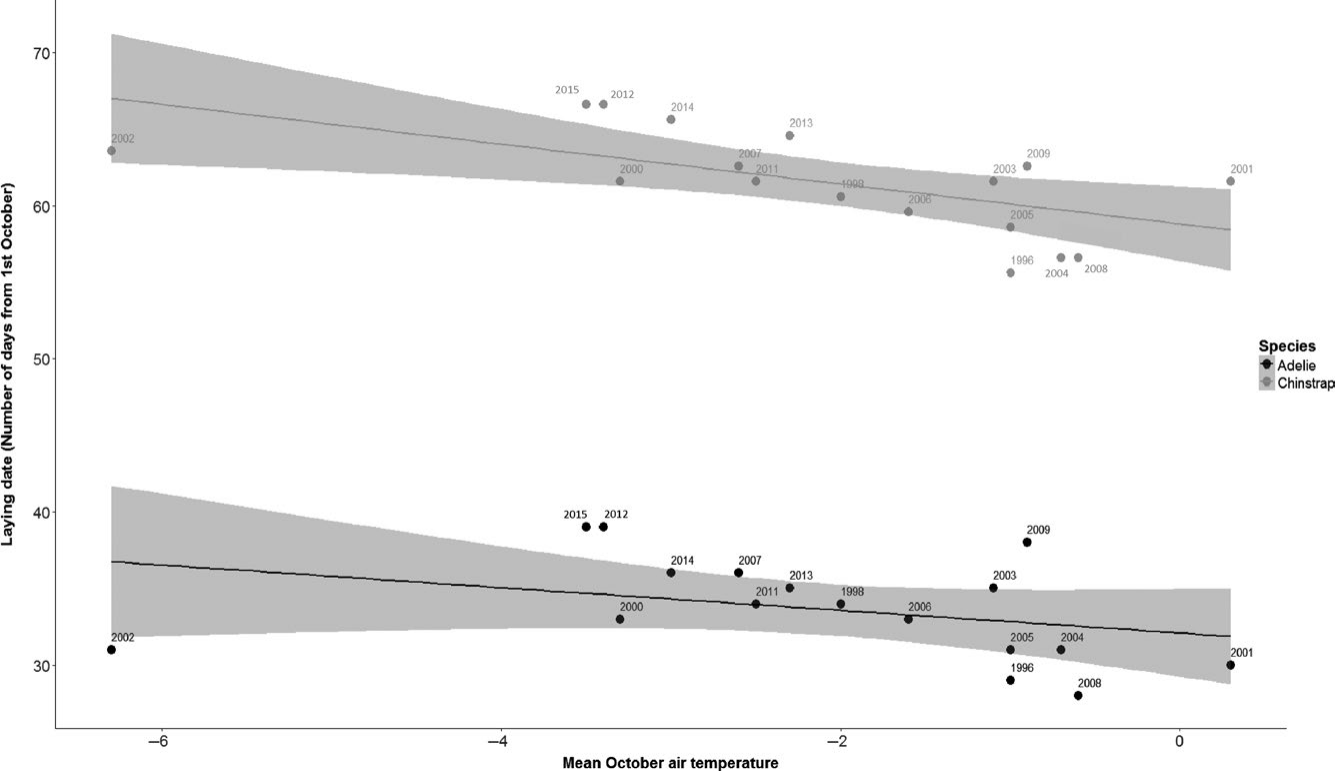
Annual laying date for Adélies (black) and chinstraps (grey) against annual mean October air temperatures (°C) over the 20‐year study period. Points are marked with years, and shading represents 95% confidence intervals

October air temperatures in the South Orkneys have increased significantly over the last 114 years from an intercept of −4.25°C ± 0.35 in 1903 at a rate of 0.017°C ± 0.005 per annum (linear regression: *F*_1,112 _= 11.28, *p* < 0.005). However, there was considerable annual variability around the trend (*SD* of model residuals = 1.87), and the adjusted *r* ^2^ showed that the time trend explained just 8.3% of the variance. There was no significant trend over the 20‐year period for which penguin phenology data were available (linear regression: *F*_1,19 _= 0.30, *p* > 0.5), although the last 5 years of the time series were among the eight coldest on record, suggesting a recent shift to cooler temperatures (Figure 5).

Modelling of the long‐term time series of phenology data revealed that the interactive effect of species and October air temperature on laying date was not significant (ANCOVA; *F*_1,30 _= 0.68, *p* > 0.4), but the slope of temperature (*F*_1,31 _= 9.04, *p* < 0.01) and difference in the intercept between the two species (*F*_1,31 _= 734.04, *p* < 0.001) were significant. Both species advanced laying dates with temperature at the same rate of 1.02 ± 0.34 days for a 1°C increase in temperature (Figure 5). The mean Adélie penguin laying date when October temperature was 0^o^ C was 1st November ± 1.02 days and that of chinstraps was 27.89 ± 1.03 days later (Figure 5). We found that the annual residuals from this model were correlated between the two species (Pearson correlation, *r* = 0.767, *t*_15_ = 4.64, *p* < 0.0005), suggesting a common phenological response to variables other than October air temperature. Allochrony was therefore conserved because the two species advanced their phenology in relation to environmental variability at the same rate. Laying dates of both species became significantly later between 1996 and 2015 at a rate of 0.37 ± 0.08 per annum (*F*_1,31 _= 20.8, *p* < 0.001) owing to the higher incidence of cool October temperatures in recent years.

## 4 DISCUSSION

Seabirds may experience high levels of interspecific competition due to their coloniality and central‐place foraging strategy (Polito et al., 2015; Rosciano et al., 2016) and reduce this by partitioning their niches along multidimensional axes such as dietary, spatial or temporal segregation (Navarro et al., 2013; Polito et al., 2015; Pratte, Robertson, & Mallory, 2017). The three species of *Pygoscelis* penguins have become a classic case study in this regard (Trivelpiece et al., 1987). Studies of spatial overlap have mostly been directed at comparing either Adélie or chinstrap penguins with gentoo penguins *Pygoscelis papua*, which occupy a distinctive niche characterised by shorter foraging ranges, deeper dives and a more fish‐based diet (Cimino, Moline, Fraser, Patterson‐Fraser, & Oliver, 2016; Kokubun et al., 2010; Miller, Kappes, Trivelpiece, & Trivelpiece, 2010). Only two have studied the spatial overlap of the ecologically similar Adélie and chinstrap penguins, both of which were confined to the chick rearing period (Lynnes et al., 2002; Wilson, 2010). Our study builds upon previous work by analysing tracking data from the entire breeding period and quantifying how allochrony gives rise to spatial segregation via leapfrog foraging. Further to this, we tested the resilience of this niche partitioning to climate change, which has the potential to alter the phenology of ecologically similar species at differing rates (Blois et al., 2013), resulting in competitor matching (Ahola et al., 2007). Reduced allochrony in response to climate change has been hypothesised to induce competitor matching among *Pygoscelis* penguins (Lynch, Fagan, et al., 2012), and our study quantifies this over a range of hypothetical and real‐world scenarios.

### 4.1 Stage‐dependent foraging distribution

We found that foraging distribution and the maximum range of trips differed significantly between breeding stages, which supports Hypothesis 1. Trips were longest during incubation compared to brood guard and tended to increase from guard to crèche for Adélie but not chinstrap penguins, as found in previous studies (Clarke, Emmerson, & Otahal, 2006; Jansen, Russell, & Meyer, 2002; Lynnes et al., 2002; Ratcliffe & Trathan, 2012). Longer incubation trips and increasing trip length with chick age are a common pattern found across seabird families (Barlow & Croxall, 2002; Ito et al., 2010; Kitaysky et al., 1999) and are related to the different energetic and time constraints that incubating eggs and feeding chicks place upon parents.

### 4.2 Allochrony and leapfrog foraging

Allochrony has long been recognised as an axis along which niche partitioning can arise for sympatric species that are otherwise ecologically similar (Birkhead & Nettleship, 1987). Adélie penguins at Signy Island initiated breeding 28 days earlier than chinstrap penguins, a degree of allochrony which is identical to another site in the South Orkneys (Carlini, Coria, Santos, & Bujan, 2005) but greater than the 21 days observed in the South Shetlands and WAP (Lynch, Fagan, et al., 2012).

The behaviour‐based model revealed that leapfrog foraging is an important mechanism for reducing foraging competition among the two species: Chinstraps performed long incubation trips while Adélies were performing short incubation and brood‐guard trips. Adélies subsequently extended their foraging ranges during crèche as chinstraps switched to short chick rearing trips for the remainder of the season. Stage‐dependent foraging ranges, combined with allochrony, therefore produced two instances of leapfrogging during the breeding season, which supports Hypothesis 2. A similar pattern of leapfrog foraging has been documented for northern and southern giant petrels *Macronectes halli* and *giganteus* (Granroth‐Wilding & Phillips, 2018) breeding sympatrically and asynchronously on South Georgia. We postulate that leapfrog foraging will arise wherever two colonial, central‐place foraging species display a combination of allochrony and stage‐dependent foraging ranges, and present 16 further examples of where this might arise for seabirds in Supporting information Table S3.

Theoretical simulations showed that if the two penguin species were to breed synchronously, their peripheral spatial overlap would increase by 54.0% over the entire breeding season, which supports Hypothesis 3. Previous studies of foraging distributions in Adélie and chinstrap penguins during chick rearing alone (Lynnes et al., 2002; Wilson, 2010) did not adequately account for the effects of allochrony and therefore overestimated the degree of spatial overlap. Previously, allochrony was shown to offset the timing of peak energetic demands associated with chick rearing for sympatric Adélie and chinstrap penguins and for Brünnich's and common guillemots *Uria lomvia* and *U. aalge* (Barrett et al., 1997; Trivelpiece et al., 1987). Our results demonstrate that allochrony can additionally reduce overlap in the foraging areas where those demands are met, further partitioning niches.

### 4.3 Partitioning of dive depths

Vertical niche partitioning has been found in a range of diving (Cimino et al., 2016; Kokubun et al., 2010, 2016; Mori & Boyd, 2004) and arboreal (MacArthur, 1958; Mansor & Ramli, 2017; Slagsvold, 1975) species where they occur in sympatry. We found that, while dive depths overlapped considerably, chinstraps dived to significantly deeper depths than Adélies. Wilson (2010) found a similar level of overlap in dive depths between these species in the South Shetland Islands, but there chinstraps dived to shallower depths than Adélies, showing that patterns of vertical partitioning among species may vary geographically. We also found evidence that the degree of overlap in dive depths was dependent on the degree of horizontal overlap in foraging areas, which supports Hypothesis 4. Vertical overlap in dive depths was reduced in core foraging areas compared to areas of peripheral or no horizontal overlap. This arose from Adélies diving on average three metres shallower in core foraging areas, which are mostly found in shallow waters close to Signy Island. Here, chinstraps are known to perform benthic dives (Takahashi et al., 2003), whereas Adélies generally rarely do so (Ropert‐Coudert et al., 2002), so it possible that Adélies perform shallower pelagic dives when foraging in shallow water with high densities of benthic‐feeding chinstraps. Similarly, Cimino et al. (2016) found that gentoo penguins performed deeper dives in areas of overlap with Adélie penguins compared to areas of no overlap, presumably to avoid competition with the shallower diving species.

### 4.4 Phenology, climate change and competitor matching

Climate change has significantly influenced species interactions and ecosystem functioning on a global scale (Cotton, 2003; Parmesan & Yohe, 2003; Visser & Both, 2005). Avian phenology is particularly sensitive to warming temperatures (Visser, te Marvelde, & Lof, 2012) and rates of change can vary among sympatric species with similar ecological requirements, resulting in competitor matching. For example, nest site competition between great tits *Parus major* and pied flycatchers *Ficedula hypoleuca* was greatest when environmental conditions synchronised their breeding phenology (Ahola et al., 2007). Analysis of long‐term monitoring data revealed that both Adélie and chinstrap penguins advanced their laying phenology at the same rate of 1.02 days per 1°C increase in October air temperature, supporting Hypothesis 5. This rate of change is lower than the rate of 1.7–1.8 found for the same two species by Lynch et al. (2012) at colonies in the South Shetlands and Western Antarctic Peninsula. Importantly, phenological responses to October air temperature and residual variability around this relationship occurred in parallel for the two species, such that allochrony was preserved in the face of environmentally induced change. Similarly, Lynch et al. (2012) found allochrony between these two species was preserved in relation to October temperature within sites though time, while Black (2015) found it was preserved across sites situated over a wide latitudinal gradient.

The ecological causes of this marked resilience of allochrony to environmental variability warrant further exploration. Adélies occur around the whole of Antarctica and only overlap with chinstraps in a small fraction of their range in the WAP and islands of the Scotia Sea (Williams, 1995). As such, avoidance of competition with chinstraps will not have been an important selective pressure upon the evolution of Adélie phenology across their range. Rather, their early phenology is believed to have evolved to allow them to exploit peaks in food availability following the spring bloom, avoid competition with migrant baleen whales and complete the breeding and moult cycle prior to the onset of the Antarctic winter (Trivelpiece et al., 1987; Youngflesh et al., 2017). Breeding success of Adélies has a tendency to be lower when laying is delayed (Hinke, Polito, Reiss, Trivelpiece, & Trivelpiece, 2012; Smiley & Emmerson, 2016; Youngflesh et al., 2017), such that there will be a selective pressure for Adélie penguins to lay as early as snow and sea ice conditions at a site allow.

Chinstrap phenology may be constrained by environmental conditions in the same way as that of Adélies, except that their phenology is delayed to a greater degree as their adaptation to the milder conditions of maritime Antarctica results in them being less cold tolerant than Adélies (Trivelpiece et al., 1987). Alternatively, chinstraps may arrive at a site and adjust their laying phenology according to the stage of the Adélies’ breeding season with the aim of minimising foraging competition. Our simulation model shows that spatial overlap in core foraging ranges increased by an average of 2.1% over the entire breeding season for each day of reduction in allochrony, which creates a strong selective pressure for chinstraps to maintain allochrony by adjusting their own breeding season relative to that of Adélie penguins. Separating these competing explanations for maintenance of allochrony will require comparisons of chinstrap phenology across multiple sites where they breed in sympatry and parapatry with Adélies.

Variation in the abundance of Antarctic krill (Ratcliffe & Trathan, 2012), both species’ primary prey, may also influence competitive interactions and thus the resilience of allochrony to environmental variability. However, current knowledge on seasonal prey abundance in this region is limited so it was not possible to investigate the role of this factor in this study.

## 5 CONCLUSIONS

Our combined analytical approach has allowed important insights into competitive interactions among the two penguin species. The behaviour‐based model reveals that niche partitioning by leapfrog foraging is reduced as the degree of allochrony between the two species is reduced, but the analysis of long‐term phenology data shows that allochrony is preserved as air temperatures warm and penguin laying dates advance. We conclude that competitor matching due to differing rates of phenological response to environmental change is unlikely to arise among the two species and will not be a significant contributing factor to the population declines observed for these two species across the WAP and Scotia Sea (Dunn et al., 2016; Lynch, Naveen, et al., 2012; Trivelpiece et al., 2011). These declines are more likely to be driven by changes in recruitment rates of Antarctic krill, recovery of the populations of other competitors such as baleen whales or direct weather effects upon penguin breeding success (Lynch, Naveen, et al., 2012; Trivelpiece et al., 2011).

## AUTHORS’ CONTRIBUTIONS

The framework for the study was developed by N.R. and H.L.C. Fieldwork was conducted by N.R., H.L.C., A.T. and S.W. with H.L.C. processing and analysing all data. H.L.C. wrote the paper with contributions from all the other authors, and all authors gave final approval for publication.

## Supporting information

** **

## Data Availability

All data presented in this paper are available from the NERC Polar Data Centre Repository: https://doi.org/10.5285/b759af06-2bb6-470e-bbac-f89fa1f940f0 (Ratcliffe & Clewlow, 2018), https://doi.org/10.5285/281e698f-0f1a-46fe-a13c-dece55f9f052 (Takahashi & Watanabe, 2018) and https://doi.org/10.5285/274b49bd-40a4-4e5d-bd5f-69a1ba09d403 (Dunn, 2018). Tracking data are additionally available from the BirdLife Seabird Tracking Database ( http://www.seabirdtracking.org/mapper/index.php).
