# Supplementary material for: Niche partitioning of sympatric penguins by leapfrog foraging appears to be resilient to climate change
Source: J Anim Ecol. 2018 Dec 3;88(2):223–35. doi: 10.1111/1365-2656.12919 (PMC7379715; doi:10.1111/1365-2656.12919)
Supplement: Supplementary file 1 [file JANE-88-223-s001.docx]

# **SUPPORTING INFORMATION**

**Appendix S1**: Sampling and variability of tracks among years

Owing to the high expense of tracking devices and the logistical challenges of maintaining field teams in Antarctica, the sample sizes of tracks within years were relatively small (Table S1), which necessitated pooling of samples across years. Furthermore, obtaining samples from all stages of the penguin breeding cycle can be challenging at Signy Island since it is a summer-only base and input and uplift dates often prevent work during the early and late season. This resulted in sample sizes being particularly small, and sampling occurring in single years, for Adélies during incubation and chinstraps during crèche (Table S1). It is important to assess how this sampling pattern might affect our conclusions about leapfrog foraging.

The small sample sizes during the Adélie incubation stage and chinstrap crèche stage does not create uncertainty in overlap statistics under the observed level of allochrony owing to the complete absence of the competing species from the focal species’ foraging range at these times of year. When Adélie penguins are incubating, all chinstraps are courting and nest building on land (Lynnes, Reid, Croxall, & Trathan, 2002), whereas when chinstraps are entering crèche, Adélies are migrating south to the Weddell Sea (Dunn, Silk, & Trathan, 2010).

To investigate annual variability in foraging patterns across years within stages we performed kernel analysis, calculated core and peripheral isopleths and plotted these for each species-stage-year combination for which data were available (for Methods see main article). Figure S1 shows that incubation trips of chinstraps had broadly similar offshore ranges and distributions during 2014 and 2016. During brood guard, ranges were relatively short during all years except for 2016 for both species, when trips extended further offshore, and 2014 for Adélies when trips were of an intermediate range. Importantly, the ranges of both species during brood guard extended in tandem during 2016, and so would not confound sampling of leapfrog foraging across species and stages in the pooled data. Crèche trips for Adélies were broadly similar in 2014 and 2016, but short in 2012, although the latter is likely due to a single trip being sampled immediately after brood guard, before the crèche stage foraging ranges had reached their full extent (Lynnes et al., 2002). Utilisation Overlap Distribution Indices for pairs of years within stages and species (for Methods see main article) confirms that foraging distributions were similar within stages across years, apart from those during brood guard during 2016 that were anomalous (Table S2).

The areas used in our study are similar to those described in a satellite tracking study of penguin foraging at Signy Island during chick-rearing in 2000 and 2001 (Lynnes et al., 2002). This study also found that Adélie penguins increased their foraging ranges from brood guard to crèche while those of chinstrap penguins remained similar during both stages. More broadly, the species-stage variability in foraging ranges, specifically Adélies performing medium-range incubation trips, short guard trips and long crèche trips and chinstraps performing long incubation trips and short guard and crèche trips, are typical of these species throughout their range (see (Clarke, Emmerson, & Otahal, 2006; Ratcliffe & Trathan, 2012) for reviews). We therefore conclude that, despite the small sample sizes for some species-stage combinations and uneven sampling across years, our results characterise the stage-specific foraging patterns that are typical of both species and our inferences about leapfrog foraging are supported.

**TABLE S1** GPS/TDR tag deployments summed by breeding stage for all years of deployment.

| **Species** | **Stage** | **Total equipped birds** | **Total foraging trips** | **Total dives** | **Sampling years (number of birds tagged)** |
| --- | --- | --- | --- | --- | --- |
| **Adélie** | Incubation | 4 | 5 | 7,799 | 2014 (4) |
|  | Guard | 33 | 44 | 30,780 | 2008(19), 2012(3), 2014(7), 2016(4) |
|  | Crèche | 12 | 18 | 28,764 | 2012(1), 2014(6), 2016(5) |
| **Chinstrap** | Incubation | 19 | 21 | 44,313 | 2014(10), 2016(9) |
|  | Guard | 60 | 89 | 34,447 | 2008(36), 2012(3), 2014(3), 2016(18) |
|  | Crèche | 4 | 7 | 2,848 | 2016(4) |

**TABLE S2** Comparison between years within breeding stages using a Utilisation Distribution Overlap Index (UDOI).

| **Stage** | **Years** | **chinstrap**  **UDOI 50** | **chinstrap**  **UDOI 95** | **Adélie**  **UDOI 50** | **Adélie**  **UDOI 95** |
| --- | --- | --- | --- | --- | --- |
| **Incubation** | 2014 - 2016 | 0.08 | 0.53 | - | - |
| **Guard** | 2008 - 2012 | 0.22 | 0.96 | 0.09 | 0.53 |
| **Guard** | 2008 - 2014 | 0.16 | 0.91 | 0.11 | 0.63 |
| **Guard** | 2008 - 2016 | 0.05 | 0.37 | 0.02 | 0.10 |
| **Guard** | 2012 - 2014 | 0.09 | 0.64 | 0.11 | 0.76 |
| **Guard** | 2012 - 2016 | 0.05 | 0.23 | 0.04 | 0.25 |
| **Guard** | 2014 - 2016 | 0.01 | 0.19 | 0.06 | 0.37 |
| **Guard** | Average of all years | 0.10 | 0.55 | 0.07 | 0.44 |
| **Crèche** | 2014 - 2016 | - | - | 0.13 | 0.62 |

**
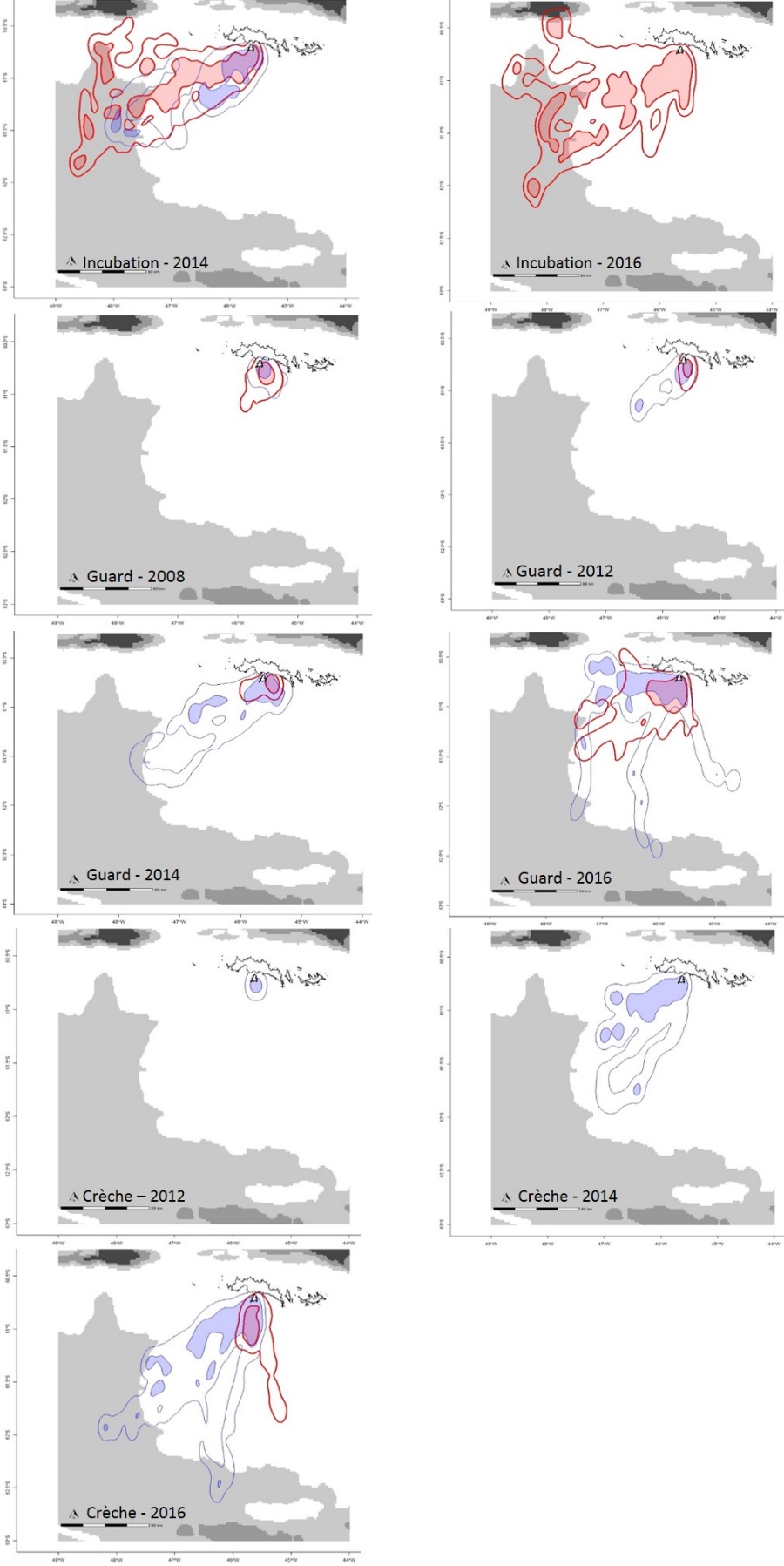
FIGURE S1** Utilization distribution kernels of peripheral (95%) (thin line) and core (50%) foraging areas (shaded area with thick line) using raw GPS data of annual stage foraging trips; incubation, guard and crèche for Adélies (blue) and Chinstraps (red) overlaid on bathymetry (metres) shown in greyscale shading. The maps were produced by the authors using R version 3.3.0.

**SUPPORTING ANIMATION S1** Animation illustrating the process of simulating tracks through the breeding season using Adélie penguins on Signy Island, South Orkneys as an example. Blue tracks represent resampled tracks from incubation, green from brood-guard and yellow from crèche.

**SUPPORTING ANIMATION S2** Animation showing overlap in 50% (shaded area with thick lines) and 95% isopleths (thin lines) of Adélie (blue) and chinstrap penguin (red) dive locations derived from the simulation analysis.

**SUPPORTING TABLE S3** Examples of allochrony in sympatric congeneric seabirds. Definitions of allochrony type: staggered allochrony is a situation where birds breed during the same season but have different peaks in laying; complete allochrony is where birds breed in different seasons (e.g. summer, winter) such that their seasons do not overlap at all; Differences in breeding synchrony is where both species overlap their breeding seasons, but one species activity is more spread out through time than the other. Leapfrog foraging may arise in those species showing staggered allochrony where this is combined with stage-dependent foraging ranges.

| **Species** | **Allochrony type** | **Broad region** | **Allochrony** |
| --- | --- | --- | --- |
| Northern (NGP) (*Macronectes halli*) and Southern Giant Petrel (SGP) (*M. giganteus*) | Staggered allochrony and differences in breeding synchrony | Polar | NGPs breed ~6 weeks earlier (Hunter, 1984) and their brooding and guarding is 11 days longer (Hunter, 1984) and display dietary partitioning (Gonzalez-Solis, Croxall, & Wood, 2000). |
| Common Murres (*Uria aalge*) and Thick-billed Murres (*U. lomvia*) | Staggered allochrony | Sub-Polar | Common murres breed ~15 days earlier and display dietary segregation between species in sympatric colonies (Barrett, Asheim, & Bakken, 1997) |
| Adélie, Chinstrap and Gentoo (*P. papua*) penguins | Staggered allochrony | Polar | Species breed at approximately 2 week intervals – Adélie followed by Gentoo, followed by Chinstrap (W. Z. Trivelpiece, Trivelpiece, & Volkman, 1987) |
| Rockhopper (*Eudyptes chrysocome*) and Macaroni (E. chrysolophus) penguins | Staggered allochrony | Sub-Polar | Dietary segregation with Macaronis eating at a higher tropic level (i.e. more fish) (Whitehead, Connan, Ropert-Coudert, & Ryan, 2017) and ~3 week difference in onset of breeding (Brown, 1987). |
| Erect-crested (*E. sclateri*) and Rockhopper penguins | Differences in breeding synchrony | Sub-tropical | Breed in sympatry in New Zealand (Williams, 1995) and display allochrony via hatching asynchrony (St. Clair, 1996). |
| Common diving (CDP) (*Pelecanoides urinatrix*) and South Georgia diving (SGDP) (*P. georgicus*) petrels | Staggered allochrony | Polar | Competition between species is reduced by SGDP utilising more barren habitats (Fischer, Debski, Taylor, & Wittmer, 2017) and the species display a ~4 week allochrony offset with CDP breeding first (Payne & Prince, 1979). |
| Wandering (*Diomedea exulans*) and Amsterdam (*D. amsterdamensis*) albatross | Complete allochrony | Sub-polar | Wandering albatross are biennial breeders and in breeding years are active between December and April, whereas Amsterdam albatross breed from March to January (Weimerskirch, Brothers, & Jouventin, 1997) |
| Black footed (*Phoebastria nigripes*) and Laysan (*P. immutabilis*) albatross | Staggered allochrony | Polar | Species display a ~2 week allochrony offset (Rice & Kenyon, 1962). |
| Black browed (*Thalassarche melanophris*) and grey headed  (*T. chrysostoma*) albatross | Staggered allochrony | Polar | Species breed ~3 weeks apart with grey headed albatross breeding first (Weimerskirch, Jouventin, & Stahl, 1986). |
| Sooty (*Phoebetria fusca*) and light mantled sooty (*P. palpebrata*) albatross | Staggered allochrony | Polar | A ~4 week allochrony offset is displayed with Sooty albatross breeding first (Weimerskirch et al., 1986). |
| Great-winged (*Pterodroma macroptera*) and white headed  (*P. lessoni*) petrel | Complete allochrony | Sub-tropical | White headed petrels breed biennially during the summer and great-winged petrels breed annually during the winter (Chastel, 1995). |
| Great-winged, Atlantic (*Pterodroma incerta*) and soft plumaged (*P. mollis*) petrel | Complete allochrony | Sub-tropical | The three species breed in sympatry on Gough Island and they have a large allochrony offset. Great-winged petrels breed in July, Atlantic petrels breed in September and soft-plumage petrels breed in December/January (Cuthbert et al., 2013; Dilley, Davies, Bond, & Ryan, 2015). |
| Murphy’s (*Pterodroma ultima*) and Kermadec (*P. neglecta*) petrel | Staggered allochrony | Tropical | Significant breeding offset with most  Kermadec petrels chicks hatching before Murphy’s petrels begin to lay (Brooke, 1995) |
| Brown (*Stercorarius antarcticus*) and south polar (S. maccormicki) skuka | Staggered allochrony | Polar | The species are sympatric for part of their ranges and in these areas they segregate their foraging niches. Brown skuas breed ~4 weeks earlier, laying at the end of November (W. Trivelpiece & Volkman, 1982). |
| Red-billed (*Phaethon aethereus*) and Yellow-billed tropicbird | Complete allochrony | Tropical | Yellow-billed tropicbirds seem to breed all year round with a peak between December and April. Whereas, red-billed tropicbirds breed in a single period between March and December (Stonehouse, 1962). |
| Crested (*Aethia cristatella*), least (*A. pusilla*) and whiskered (*A. pygmaea*) auklet | Differences in breeding synchrony | Sub-tropical | All three species arrive at the breeding colonies and begin laying at a similar time but display allochrony in their incubation periods, with Crested auklets having the longest (Knudtson & Byrd, 1982) |

# **References**

Barrett, R. T., Asheim, M., & Bakken, V. (1997). Ecological relationships between two sympatric congeneric species, Common Murres and Thick-billed Murres, Uria aalge and U. lomvia, breeding in the Barents Sea. *Canadian Journal of Zoology*, *75*(4), 618–631. doi:10.1139/z97-077

Brooke, M. de L. (1995). The breeding biology of the gadfly petrels Pterodroma spp. of the Pitcairn Islands: characteristics, population sizes and controls. *Biological Journal of the Linnean Society*, *56*(1–2), 213–231. doi:10.1111/j.1095-8312.1995.tb01086.x

Brown, C. R. (1987). Traveling Speed and Foraging Range of Macaroni and Rockhopper Penguins at Marion Island (Velocidad de Movimiento y Extensión de las Áreas de Forrajeo de los Pingüinos Eudyptes chrysolophus y E. chrysocome). *Journal of Field Ornithology*, *58*(2), 118–125.

Chastel, O. (1995). Influence of reproductive success on breeding frequency in four southern petrels. *Ibis*, *137*(3), 360–363. doi:10.1111/j.1474-919X.1995.tb08033.x

Clarke, J., Emmerson, L. M., & Otahal, P. (2006). Environmental conditions and life history constraints determine foraging range in breeding Adélie penguins. *Marine Ecology Progress Series*, *310*, 247–261.

Cuthbert, R. J., Louw, H., Lurling, J., Parker, G., Rexer-Huber, K., Sommer, E., … Ryan, P. G. (2013). Low burrow occupancy and breeding success of burrowing petrels at Gough Island: a consequence of mouse predation. *Bird Conservation International*, *23*(2), 113–124. doi:10.1017/S0959270912000494

Dilley, B. J., Davies, D., Bond, A. L., & Ryan, P. G. (2015). Effects of mouse predation on burrowing petrel chicks at Gough Island. *Antarctic Science*, *27*(6), 543–553. doi:10.1017/S0954102015000279

Dunn, M. J., Silk, J. R. D., & Trathan, P. N. (2010). Post-breeding dispersal of Adélie penguins ( *Pygoscelis adeliae* ) nesting at Signy Island, South Orkney Islands. *Polar Biology*, *34*(2), 205–214. doi:10.1007/s00300-010-0870-4

Fischer, J. H., Debski, I., Taylor, G. A., & Wittmer, H. U. (2017). Nest site selection of South Georgia Diving-petrels Pelecanoides georgicus on Codfish Island, New Zealand: implications for conservation management. *Bird Conservation International*, 1–12. doi:10.1017/S0959270917000041

Gonzalez-Solis, J., Croxall, J. P., & Wood, A. G. (2000). Foraging partitioning between giant petrels Macronectes spp. and its relationship with breeding population changes at Bird Island, South Georgia. *Marine Ecology. Progress Series*, *204*, 279–288.

Hunter, S. (1984). Breeding biology and population dynamics of giant petrels Macronectes at South Georgia (Aves: Procellariiformes). *Journal of Zoology*, *203*(4), 441–460. doi:10.1111/j.1469-7998.1984.tb02343.x

Knudtson, E. P., & Byrd, G. V. (1982). Breeding Biology of Crested, Least, and Whiskered Auklets on Buldir Island, Alaska. *The Condor*, *84*(2), 197–202. doi:10.2307/1367671

Lynnes, A., Reid, K., Croxall, J., & Trathan, P. (2002). Conflict or co-existence? Foraging distribution and competition for prey between Adélie and chinstrap penguins. *Marine Biology*, *141*(6), 1165–1174. doi:10.1007/s00227-002-0899-1

Payne, M. R., & Prince, P. A. (1979). Identification and breeding biology of the diving petrels Pelecanoides georgicus and P. urinatrix exsul at South Georgia. *New Zealand Journal of Zoology*, *6*(2), 299–318. doi:10.1080/03014223.1979.10428368

Ratcliffe, N., & Trathan, P. (2012). A review of the diet and at-sea distribution of penguins breeding within the CAMLR Convention Area. *CCAMLR Science*, *19*, 75–114.

Rice, D. W., & Kenyon, K. W. (1962). Breeding Cycles and Behavior of Laysan and Black-Footed Albatrosses. *The Auk*, *79*, 517–567.

St. Clair, C. C. (1996). Multiple Mechanisms of Reversed Hatching Asynchrony in Rockhopper Penguins. *Journal of Animal Ecology*, *65*(4), 485–494. doi:10.2307/5783

Stonehouse, B. (1962). The Tropic Birds (genusphaethon) of Ascension Island. *Ibis*, *103B*(2), 124–161. doi:10.1111/j.1474-919X.1962.tb07242.x

Trivelpiece, W., & Volkman, N. J. (1982). Feeding Strategies of Sympatric South Polar Catharacta Maccormicki and Brown Skuas C. Lönnbergi. *Ibis*, *124*(1), 50–54. doi:10.1111/j.1474-919X.1982.tb03740.x

Trivelpiece, W. Z., Trivelpiece, S. G., & Volkman, N. J. (1987). Ecological Segregation of Adelie, Gentoo, and Chinstrap Penguins at King George Island, Antarctica. *Ecology*, *68*(2), 351–361.

Weimerskirch, H., Brothers, N., & Jouventin, P. (1997). Population dynamics of wandering albatross Diomedea exulans and Amsterdam albatross D. amsterdamensis in the Indian Ocean and their relationships with long-line fisheries: Conservation implications. *Biological Conservation*, *79*(2), 257–270. doi:10.1016/S0006-3207(96)00084-5

Weimerskirch, H., Jouventin, P., & Stahl, J. C. (1986). Comparative ecology of the six albatross species breeding on the Crozet Islands. *Ibis*, *128*(2), 195–213. doi:10.1111/j.1474-919X.1986.tb02669.x

Whitehead, T. O., Connan, M., Ropert-Coudert, Y., & Ryan, P. G. (2017). Subtle but significant segregation in the feeding ecology of sympatric penguins during the critical pre-moult period. *Marine Ecology Progress Series*, *565*, 227–236. doi:10.3354/meps12017

Williams, T. (1995). *The Penguins: Spheniscidae*. Oxford University Press.
